# Femoral neck strain prediction during level walking using a combined musculoskeletal and finite element model approach

**DOI:** 10.1371/journal.pone.0245121

**Published:** 2021-02-01

**Authors:** Zainab Altai, Erica Montefiori, Bart van Veen, Margaret A. Paggiosi, Eugene V. McCloskey, Marco Viceconti, Claudia Mazzà, Xinshan Li

**Affiliations:** 1 Department of Mechanical Engineering, University of Sheffield, Sheffield, United Kingdom; 2 Insigneo Institute for in silico Medicine, University of Sheffield, Sheffield, United Kingdom; 3 Department of Oncology and Metabolism, Mellanby Centre for Bone Research, University of Sheffield, Sheffield, United Kingdom; 4 Department of Industrial Engineering, Alma Mater Studiorum, University of Bologna, Bologna, Italy; 5 Laboratorio di Tecnologia Medica, IRCCS Istituto Ortopedico Rizzoli, Bologna, Italy; North Carolina State University, UNITED STATES

## Abstract

Recently, coupled musculoskeletal-finite element modelling approaches have emerged as a way to investigate femoral neck loading during various daily activities. Combining personalised gait data with finite element models will not only allow us to study changes in motion/movement, but also their effects on critical internal structures, such as the femur. However, previous studies have been hampered by the small sample size and the lack of fully personalised data in order to construct the coupled model. Therefore, the aim of this study was to build a pipeline for a fully personalised multiscale (body-organ level) model to investigate the strain levels at the femoral neck during a normal gait cycle. Five postmenopausal women were included in this study. The CT and MRI scans of the lower limb, and gait data were collected for all participants. Muscle forces derived from the body level musculoskeletal models were used as boundary constraints on the finite element femur models. Principal strains were estimated at the femoral neck region during a full gait cycle. Considerable variation was found in the predicted peak strain among individuals with mean peak first principal strain of 0.24% ± 0.11% and mean third principal strain of -0.29% ± 0.24%. For four individuals, two overall peaks of the maximum strains were found to occur when both feet were in contact with the floor, while one individual had one peak at the toe-off phase. Both the joint contact forces and the muscular forces were found to substantially influence the loading at the femoral neck. A higher correlation was found between the predicted peak strains and the gluteus medius (R^2^ ranged between 0.95 and 0.99) than the hip joint contact forces (R^2^ ranged between 0.63 and 0.96). Therefore, the current findings suggest that personal variations are substantial, and hence it is important to consider multiple subjects before deriving general conclusions for a target population.

## 1 Introduction

In order to understand the relationship between skeleton health and mechanical loading during normal daily activities such as walking, an accurate estimation of the physiological strain distribution in the femur is essential. The mechanical response of the femoral neck under physiological loading can also be used for clinical diagnoses of potential bone and joint diseases, design of treatments (e.g. hip implants), and to optimize the performance of the treatment, especially when fully personalised muscle and bone anatomy is considered.

Finite element analysis based on computed tomography scans (FE/CT, or biomechanical CT analysis), well-established methods in the field of biomechanics [1], are commonly used to investigate the mechanical response (e.g. stress and strains) of bone to external loads. However, the loads used in these studies are mostly either arbitrary (such as those obtained from bone strength estimation studies) [2–4], or comparable to those used in particular mechanical experiments for validation purposes [5, 6], often with bone failure being the end goal. These studies are often focused on one level (organ level), where bone structural properties are investigated in details. However, there is a need to consider realistic loading parameters if the actual response of a healthy or a diseased bone is desirable under a specific daily activity or exercise. This can be achieved by considering the actual personalised joint contact and muscle forces in the designated FE models.

Musculoskeletal models (MSKM) are commonly used to evaluate joint contact and muscle forces during various dynamic tasks [7–11]. Traditionally, such studies have focused on body level, where the whole body is modelled using multiple segments to represent bones and joints (multibody dynamic models), with muscle information obtained from MRI scans.

In theory, the finite element modelling approach and MSKMs can be integrated in order to investigate the effect of a real life event with more accurate boundary conditions [12], using joint and muscle forces estimated from the latter [9, 13]. A few previous studies have attempted to use this multiscale modelling approach to investigate the strain distribution within the femoral shaft [14–19] or at the femoral neck [19–22] during various daily activities. However, numerous challenges and limitations were described in these approaches.

The majority of previous studies have used finite element models of cadaveric bones combined with gait data from body-matched volunteers [14, 16, 20]. Simulating real life daily activities, such as walking, using muscle and joint forces estimated by a musculoskeletal model of a volunteer and applied on a finite element model of the femur of another person is not truly personalised modelling, even if body-matched factors are considered. This could induce errors in the predicted strain [20]. Furthermore, the gait data have been mostly collected from young volunteers which might differ from those presented in older adults. Older adults have been reported to have more conservative gait patterns characterised by reduced velocity, shorter step length and increased step timing variability [23], lower muscle activation [24], and lower ground reaction forces, in particular the push-off phase [25]. Therefore, it is essential to utilize personalised body and organ data in order to make these simulations as physiologically relevant as possible.

Although there has been a trend towards more personalised models in recent years [19, 22], the ability of developing a fully personalised model was limited by the availability of the datasets. Kersh et al. [22] investigated the strain distribution in the proximal femur during various locomotor tasks using musculoskeletal-finite element models for twenty women. Even though they considered the gait data of the subjects, a generic musculoskeletal model was scaled for each subject and then used to evaluate individual muscle contributions to bone strain estimated by the finite element models of the subjects.

The majority of musculoskeletal-finite element modelling studies reported in the literature were limited by a small number of samples (often single anatomical datasets) [14, 16, 19, 20]. Consequently, there is a lack of understanding in the variability of bone and joint forces due to different anatomies [20]. The ability to explore intra-personal variations within multiple subjects is necessary to investigate how individual anatomical parameters, motion patterns, and other factors (such as age and weight) affect the bone and joint force estimations [26], and subsequently how these would influence the predicted strain patterns on the femur, when combined with an individual specific organ level finite element model of the bone.

Considering the fact that there is no current method to directly measure the femoral neck strain *in vivo* when performing daily tasks, a combined musculoskeletal-finite element modelling approach is well suited to provide a more accurate picture of the mechanical response of the femur during such activities. Moreover, such modelling approach can in future, help to optimize clinical decision making through reliably predicting various patient-specific parameters (such as bone strength and joint load) using non-invasive medical imaging and gait data [9, 27].

Therefore, the aim of the current study is to report the first fully personalised multiscale (body-organ level) model in order to investigate the strain distribution predicted at the femoral neck during level walking for a full gait cycle. Five subjects will be investigated in this study to compare intra-personal variations in the predicted strain patterns.

## 2 Materials and methods

### 2.1 Cohort

Five postmenopausal women (68±5 y.o., 70±7 kg, 159±4 cm) were included in this study (Table 1). Patients with osteopenia/osteoporosis presented to the Metabolic Bone Centre, Northern General Hospital in Sheffield, UK between 27 March 2017 and 14 May 2018. Willing and eligible volunteers were then recruited as participants of the Multisim Study. The study was approved by the Health Research Authority of East of England (Cambridgeshire and Hertfordshire Research Ethics Committee, reference 16/EE/0049). Written informed consents were obtained from all individuals. These patients attended the hospital on one occasion. During the visit, CT and MRI scans of the lower limbs of each case were collected. Participants underwent a 3D gait analysis on the same day of the MRI scan.

**Table 1:** Demographic of the study cases.

Case ID	Age (year)	Height (cm)	BW (kg)	BMI	BMD* (g/cm^2)	T-score*
1	70.5	164	61.4	22.8	0.604	-2.2
2	64.1	156	75.8	31.1	0.721	-1.2
3	73.0	161	78.6	30.5	0.719	-1.2
4	72.2	160	66.3	25.9	0.640	-1.9
5	61.8	155	67.0	27.9	0.603	-2.2
Mean	68.3	159	69.8	27.7	0.657	-1.7
SD	5.05	3.65	7.14	3.42	0.060	0.5

BW, body weight

*Values for femoral neck as determined by dual energy x-ray absorptiometry (DXA) using a Discovery A densitometer (Hologic Inc., Bedford, MA, USA).

Exclusion criteria were: body mass index (BMI) <18 or >35, history of or current conditions known to affect bone metabolism and bone mineral density, history of or current neurological disorders, prescription of oral corticosteroids for more than three months within the last year, history of any long term immobilization (>3 months), conditions that prevent the acquisition of musculoskeletal images, use of medications or treatment known to affect bone metabolism other than calcium/vitamin D supplementation and alcohol intake greater than 21 units per week [28].

### 2.2 Geometrical parameters

**Fig 1:**
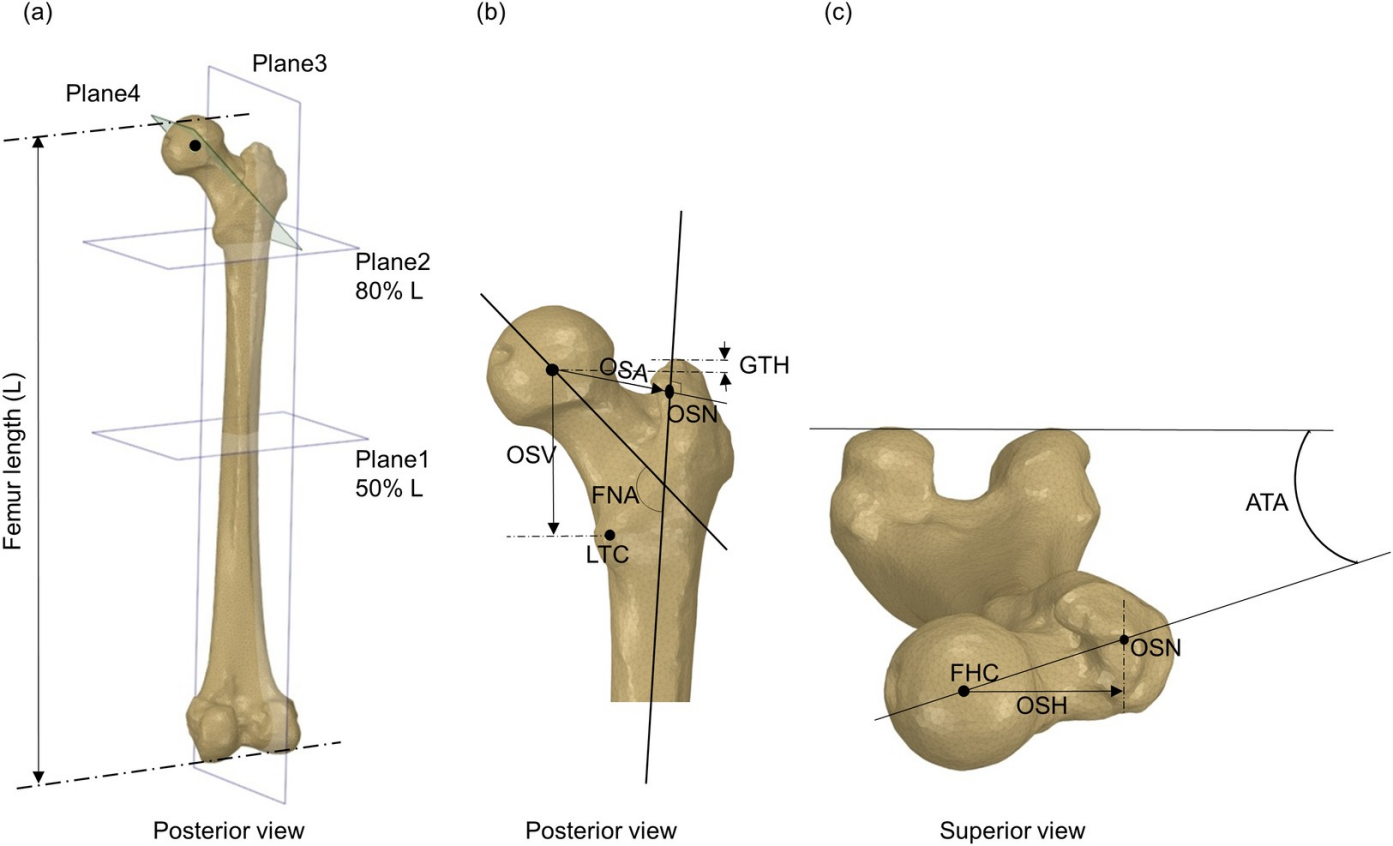
Schematic drawing illustrating the measured morphological parameters of the femur. Definition of each parameter is described in Table 2. (a) and (b): posterior view, (c): superior view of a femur.

The morphological parameters of the full femur were investigated in this study following the method described by Soodmand et al. [29]. For the five cases, the 3D geometry of the right femur was segmented from the CT scans using Mimics 20.0 (Materialise, Belgium). The segmented femora were later used to develop finite element models. Measurements were taken on the segmented 3D geometries of the femoral bones using SpaceClaim 19.1, (ANSYS Inc., PA, USA). For each femur, four planes were defined as shown in Fig 1(A). Plane 1 and Plane 2 are transversal planes at 50% and 80% of the total length of the femur (L) with respect to the distal end of the femur. Plane 3 is a sagittal plane passing through the femoral shaft and includes the midpoints of the shaft cross sections at the 50% and 80% planes. Femoral shaft axis was defined as the axis passing through these two mid points in Plane 3. While Plane 4 is a plane passing through the centre line of the femoral neck and contains the centre of the femoral head. The femoral neck axis was defined as the axis passing along the femoral neck centre line in the frontal plane and includes the centre of femoral head.

**Table 2:** Definition of the measured morphological parameters.

Parameter	Definition
Femoral head centre (FHC)	The centre of best fitted sphere at the femoral head
Femoral head diameter (FHD)	The diameter of best-fit sphere at the femoral head
Total femur length (L)	The distance between the most distal and the most proximal point of the femur in the longitudinal direction
Femoral neck angle (FNA)	Angle made by axis of femoral shaft in Plane 3 and the line passes through the centre of the femoral head along the axis of the femoral neck in Plane 4
Offset intersection node (OSN)	The intersection point of the femoral shaft axis and femoral neck axis
Absolute offset (OSA)	Absolute distance between femoral head centre and mid shaft axis
Horizontal offset (OSH)	Horizontal distance between femoral head centre and mid shaft axis
Lesser trochanter centre (LTC)	The centre of best fitted sphere at the lesser trochanter
Vertical offset (OSV)	Vertical distance between the centres of the femoral head and the lesser trochanter
Greater trochanter height (GTH)	Vertical distance between femoral head centre and the most proximal node at the greater trochanter. GTH with plus sign means GTH node is above the femoral head centre, while GTH with minus sign means GTH node is below the femoral head centre
Anteversion angle (ATA)	Angle between the transverse line passing through the femoral head centre and the neck centre line and an imaginary line passing through the medial and lateral condyles

Description of the morphological parameters measured for the femur of the five cases included in the current study and adapted from Soodmand et al. [29].

Selective morphological parameters were chosen to investigate the geometrical variation of the femoral neck (the region of interest) of the five cases included in this study. All parameters are illustrated in Fig 1 and described in Table 2.

### 2.3 Musculoskeletal modelling

#### 2.3.1 Gait analysis

For the gait analysis, participants were asked to walk barefoot along a 10 m walkway at a self-selected walking speed. Marker trajectories from five valid walking trials per participant were recorded at 100 Hz using a 12-camera motion capture system (Vicon, Oxford, UK). A modified Vicon Plug-in-Gait marker set was used. Ground reaction forces were simultaneously acquired at 1000 Hz using two force platforms (Kistler, Winterthur, Switzerland), allowing to record forces for one full stride. Vicon Nexus was used to label marker trajectories and fill gaps <5 frames. Prior to the MRI scans, the position of several motion capture markers was drawn on the skin to allow exact replacement with MRI-visible markers during the scan. The markers’ location was then used in the model for the registration of MRI and gait analysis data.

#### 2.3.2 Magnetic resonance imaging

Full lower limb MRI was collected using a Magnetom Avanto 1.5 T scanner (Siemens, Erlangen Germany). A T1-weighted scanning sequence was used with an echo time of 2.59 ms, a repetition time of 7.64 ms, flip angle of 10 degrees and voxel sizes of 1.1x1.1x5.0 mm for the long bones and 1.1x1.1x3.0 mm for the joints. Within the MRI scans, all lower limb bones were segmented using Mimics 20.0 (Materialise, Leuven, Belgium).

In each limb, 23 muscles were segmented, which are listed in Table 3. Initially these muscles were segmented using the automated muscle segmentation toolbox (Mimics Research 20.0, Materialise, Belgium), after which manual adjustments were performed when necessary. Data were all processed by the same expert operator, and intra-operator repeatability of the procedure was ensured by calculating the volume (V_M_) of the segmented muscles over three repetitions. Bone and muscle segmentations have been made available as part of a previous publication through Figshare (https://doi.org/10.15131/shef.data.9934055.v3).

**Table 3:** List of muscles included in the musculoskeletal model.

Model muscles	Segmented	FE Model
Adductor Brevis	✔	✔
Adductor Longis	✔	✔
Adductor Magnus	✔	✔
Biceps Femoris long head	✔	✔
Biceps Femoris short head	✔	✔
Extensor Digitorum		
Extensor Hallucis		
Flexor Digitorum		
Flexor Hallucis		
Gemellus		✔
Gluteus Maximus	✔	✔
Gluteus Medius	✔	✔
Gluteus Minimus		✔
Gracilis	✔	
Iliacus	✔	✔
Gastrocnemius Lateralis	✔	✔
Gastrocnemius Medialis	✔	✔
Pectineus		✔
Peroneus Brevis	✔	
Peroneus Longus		
Peroneus Tertius		
Piriformis		✔
Psoas		✔
Quadratus Femoris		✔
Rectus Femoris	✔	
Sartorius	✔	
Semimembranosus	✔	
Semitendinosus	✔	
Soleus	✔	
Tensor Fasciae Latae	✔	
Tibialis Anterior	✔	
Tibialis Posterior	✔	
Vastus Intermedius	✔	✔
Vastus Lateralis	✔	✔
Vastus Medialis	✔	✔

The forces produced by the muscles attached to the femur were calculated and applied to the finite element model (details are described in section 2.3.3).

#### 2.3.3 Musculoskeletal models (MSKM)

The MSKM was created from the segmented bone geometries of the MRI scans and included seven body segments (pelvis, two femora, two tibiae, two feet) articulated by six joints: an ideal ball-and-socket joint for the hip, and two ideal hinges for knee and ankle [30, 31]. The muscles included in the gait2392 generic model [32] were added to the model by directly identifying their origin and, insertion points via MRI.

Musculotendon (MT) units were modelled using a three element Hill-type muscle model, requiring the definition of the following five MT parameters: optimal fibre length (l_opt_), tendon slack length (l_TS_), pennation angle, maximal contraction velocity and maximal isometric force (F_max_). The l_opt_ and l_TS_ were scaled to maintain the l_opt_/l_MT_ and l_TS_/l_MT_ ratios of the gait2392 model (l_MT_ = musculotendon length). The pennation angle was set according to the gait2392 model and the maximal contraction velocity was set to 10 fibres per second. For the 23 segmented muscles, values of F_max_ were calculated on the basis of the relationship with physiological cross-sectional area (PCSA) [33] using segmented muscle volumes according to Eq (1):

$$F_{max}=k*\frac{V_{M}}{l_{opt}}$$ (1)

where k is the specific tension (61 N/cm^2^ [34]), V_M_ is the volume of the segmented muscle, and V_M_/l_opt_ corresponds to the muscle PCSA.

For the remaining muscles, F_max_ was linearly scaled to the lower-limb mass from the gait2392 model according to (2):

$$F_{max}=\frac{m_{LL}}{m_{LLGen}}*F_{maxGen}$$ (2)

where m_LL_ is the mass of the lower limb of the subject, calculated as a product of the volume of the lower limb (estimated from the MRI) and the density of the tissue [35], m_LLGen_ is the mass of the lower limb of the gait2392 model and F_maxGen_ is the default F_max_ of the muscles in the gait2392 model. Eighteen muscles, those that are directly attached to the femur, were selected to be considered in the finite element model (Table 3). The full gait cycle was divided into one hundred intervals. At each interval muscle forces were estimated and then applied to the finite element models.

#### 2.3.4 Dynamic simulations

Joint angles and moments were computed within OpenSim 3.3 [9] using the MATLAB API (v9.1, R2017b, Mathworks, USA) and the Inverse Kinematics (marker weights set to 1 for all the markers) and Inverse Dynamics (coordinates filtered at 6 Hz) tools, following the OpenSim good practice recommendations [27]. Static Optimisation was then run minimizing the sum of muscle activations squared [36] and neglecting the force-length-velocity (FLV) relationship of the muscles to compute their force and activation. This choice was pursued for all the models and simulations. Ideal moment generators (reserve actuators), providing joint torque when muscle forces could not balance the external moments, were included for each degree of freedom, but made unfavourable to recruit by assigning them a unitary maximum force. Musculoskeletal models did not include an upper body due to the missing MRI data and hence the inertial properties couldn’t be determined as with the lower limb segments. Therefore, the residual reduction algorithm (RRA) has not been used. Finally, Joint Reaction Analysis [37] was run to calculate joint contact forces (JCFs).

Hip and knee joint contact forces were then used to check that the boundary conditions imposed on the finite element model were appropriate and statically equivalent to applying the hip and knee joint reaction forces.

### 2.4 Finite element models (FEM)

#### 2.4.1 Finite element model construction

**Fig 2:**
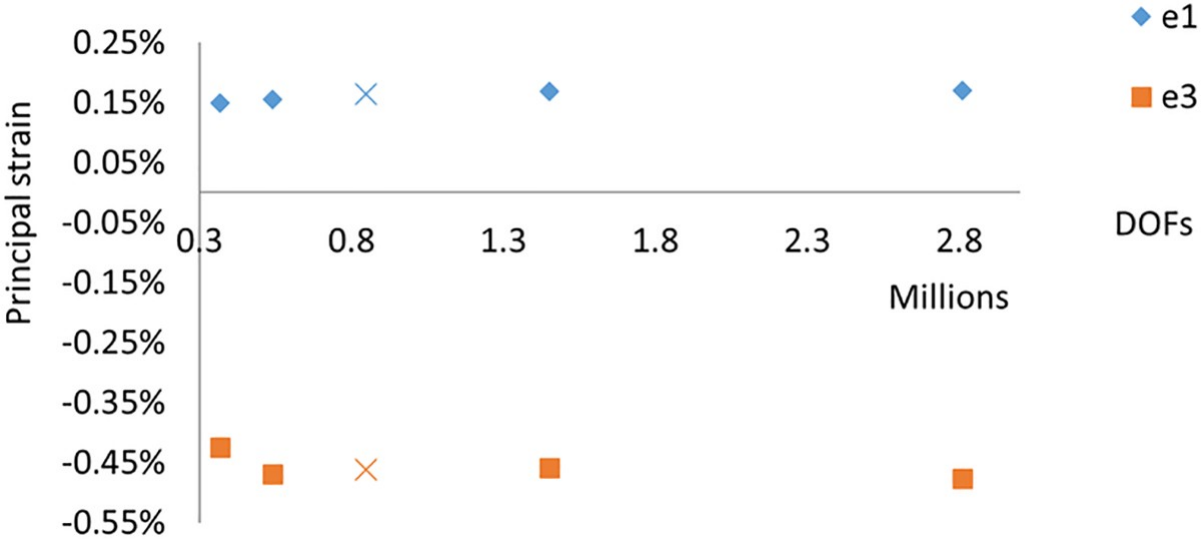
Mesh convergence study. Four different element sizes (2, 2.5, 3, 3.5, 4 mm) were tested. The mesh was converged at the 3 mm element size (highlighted above as ×). This element size was used for all five subjects in the final finite element analysis. DOF = degrees of freedom.

The finite element models of the full femur were created from the CT scans (tube current:120 mA, tube voltage: 100 kVP, and a resolution of 0.742X0.742X0.625 mm^3^) using a GE scanner (LightSpeed 64 VCT, GE Medical Systems, Milwaukee, WI, USA). The full femur of the right limb for the five cases was segmented in Mimics 20.0 (Materialise, Belgium). The segmented femora were then automatically meshed with 10-node tetrahedral element type (ICEM CFD 15.0, ANSYS Inc., PA, USA) with an average element size of 3mm. A mesh convergence study was conducted using four different element sizes (2, 2.5, 3, 3.5, 4 mm) on one subject (Case 2) as shown in Fig 2. The values of first (e1) and third (e3) principal strains changed by only 4% and 3%, respectively, within the volume of interest (femoral neck) between the selected mesh refinement (3 mm) of 849,069 degrees of freedom (DOF) and the finest mesh (2 mm) of 2810736 degrees of freedom. Bone materials were defined as linear elastic isotropic. Heterogeneous material properties were estimated from the CT scan and mapped to the finite element models following a well-validated material-mapping procedure (Bonemat v3, Rizzoli Institute) [38–40]. ESP phantom was used for bone density calibration.

#### 2.4.2 Load application and boundary conditions

**Fig 3:**
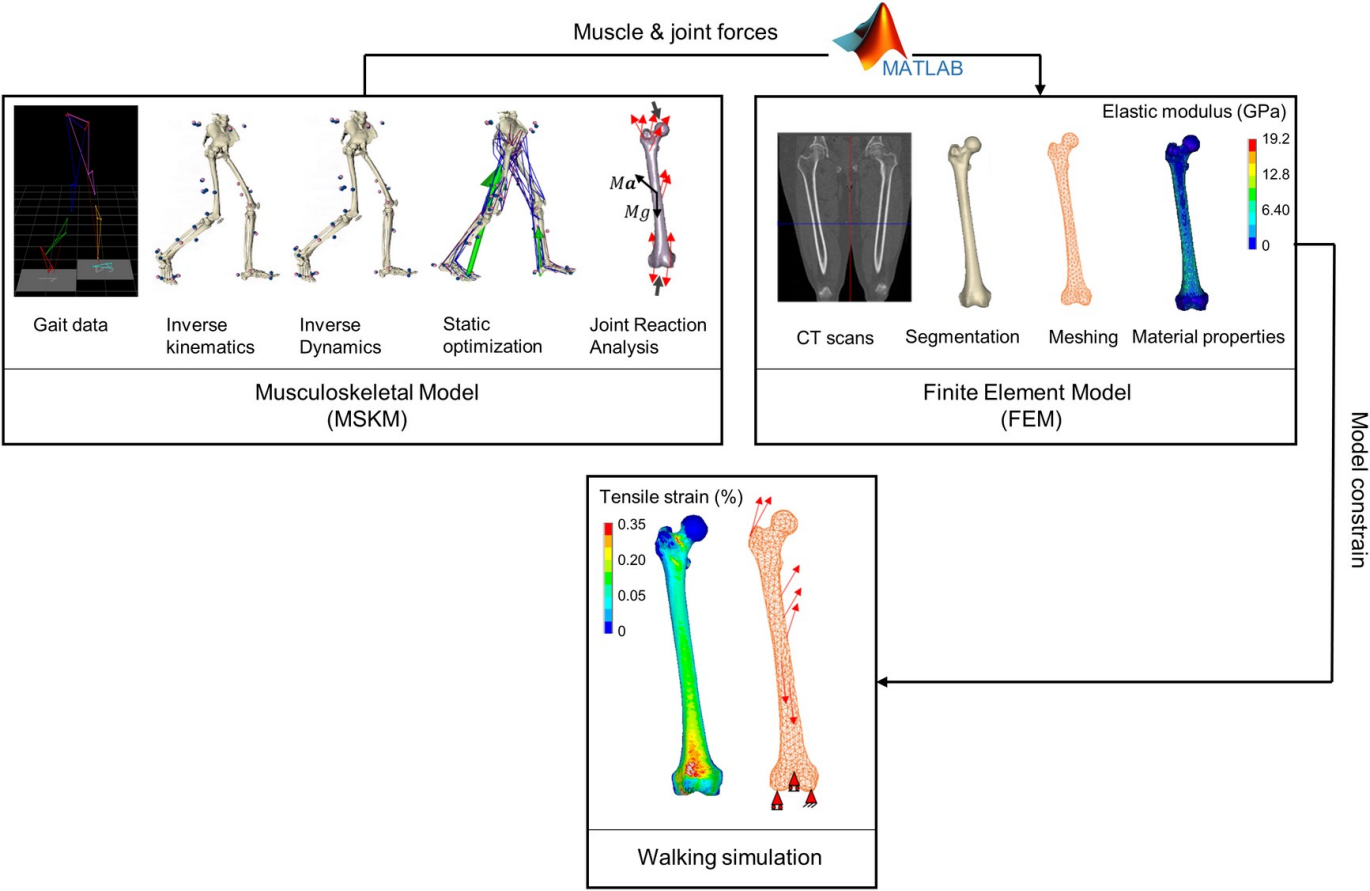
Multiscale modelling workflow. Diagram illustrates the various steps of the multiscale modelling workflow followed in this study: musculoskeletal modelling (top left), CT based finite element modelling (top right), and body-organ coupling (bottom) by applying the muscle and joint forces to the finite element model.

For each case, muscle forces calculated by the musculoskeletal model, as described in section 2.3, were applied to the finite element model in order to investigate the strain produced on the femoral neck during a normal gait cycle using a fully personalised multiscale model. Fig 3 illustrates the multiscale modelling approach followed in this study. For a feasible body-organ coupling, the force balance of the musculoskeletal model could be simplified in the form represented in Eq (3):

$$Ma=\sum F_{muscle}+\sum F_{joint}$$ (3)

Where *M* is the body mass, ***a*** is the acceleration, ***F***_*muscle*_ is the muscle force, and ***F***_*joint*_ is the joint contact force. As the assumption was to perform quasi-static simulation using FEM in order to represent a snap shot of the position during the gait cycle, the inertial and acceleration forces were set to zero, leading to Eq (4):

$$0=\sum F_{muscle}+\sum F_{joint}$$ (4)

However, in reality there are likely to be numerical errors accumulated in both the musculoskeletal model and the finite element model, as well as the assumption to conduct quasi-static analysis through the gait cycle. Hence, Eq (4) was further modified to Eq (5), where *δ* represents the residual forces, as a result of the computational errors. The value of *δ* should be relatively small.

$$\delta =\sum F_{muscle}+\sum F_{joint}$$ (5)

All muscle and joint forces were transformed from the MRI to the CT scans reference frame. The Iterative Closet Point (ICP) algorithm [41] was used in Matlab (R2019a) to apply the rotation and the translation matrix for optimal fitting. The mean of the root squared error (of the transformation) across all patients were 2.08±0.70 mm.

The eighteen muscle forces were applied to the finite element model as point loads at the external surface of the femur. The location of the attachment point of each muscle was estimated by the musculoskeletal model and used to allocate the point of application of the force in the finite element model. Forces were then applied at the closest surface mesh node to the point of application estimated by the musculoskeletal model. The distance between the point of application of the forces estimated by the musculoskeletal model and the closest nodes in the finite element model varied between 0.1 to 1.6 mm with an average value of 0.91±0.36 mm.

The finite element models were kinematically constrained at the distal end of the femur to prevent rigid body motion. These constrains were chosen so that the equilibrium of the forces estimated by the MSK model was not disturbed, in other words, to get the residual values as small as possible or close to zero as shown in Eq (5). Two different boundary conditions for the finite element model were tested: (a) the distal end of the femur was totally fixed, which represented the most constrained condition that can produce the highest reaction forces; and (b) a more relaxed BCs at the distal end by constraining only three selected nodes as follow; the most distal node of the medial condyle was completely fixed, while the displacement of the most distal node at the lateral condyle was constrained in the anterior-posterior and vertical (superior-inferior) directions. A third node in the patella groove was constrained antero-posteriorly [15]. These boundary conditions were chosen to replicate the basic movements involved in walking, which are flexion-extension and rotation at the hip, knee and ankle joints; abduction-adduction predominantly at the hip joint [42]. The residuals and predicted strains were then evaluated for both boundary constraints. The relaxed boundary constraint (b) produced the least residuals, whereas only very small differences were found in the predicted peak principal strains of the first and the second boundary conditions (1.6% and 0.8%, respectively). Hence, the amore relaxed boundary condition was chosen. The boundary conditions were statically equivalent to applying the appropriate hip and joint contact forces (details are in the results section).

#### 2.4.3 Quasi-static simulations

To understand thoroughly the strains produced on the femoral neck during a normal gait cycle, the full gait cycle was discretised into one hundred even intervals. At each interval, the peak first and third principal strains at the femoral neck were averaged across the surface nodes using a circle of 3mm radius, to follow the continuum hypothesis avoiding local effects of the load [3, 43]. The peak predicted strains were then compared to the elastic limit of the human bone (0.73% and 1.04% for tensile and compressive strain, respectively) [44]. The location of the peak strains within the femoral neck region was also analysed. The relation between the individual femoral forces, including the joint and muscle forces, and the predicted strains was evaluated using linear regression analysis. Furthermore, the major muscles affecting the bulk femoral neck loading were also investigated. All simulations were performed in a work station using ANSYS Mechanical APDL 19.1 (Ansys Inc., PA, USA). The computing time was around 60 seconds for each interval of the gait cycle.

## 3 Results

The measured morphological parameters for the five femora investigated in the current study are listed in Table 4. The maximum standard deviation (SD) was observed in the measurement of vertical offset (OSV) of 10.38 mm, while the minimum SD was found in the measurement of femoral head diameter (FHD) of 1.07 mm.

**Table 4:** Morphological parameters measured for the five femora.

Case ID	FHD (mm)	L (mm)	FNA (degree)	OAS (mm)	OSH (mm)	OSV (mm)	GTH (mm)	ATA (mm)
1	43.88	426.50	128.50	31.90	31.90	50.20	-1.20	11.60
2	42.00	424.60	132.90	34.60	33.70	60.06	-4.50	12.93
3	41.40	410.60	123.30	40.40	35.90	49.48	4.50	8.19
4	44.00	439.42	132.30	36.80	32.16	57.30	3.10	9.95
5	43.60	415.80	126.10	40.75	40.60	30.36	2.30	9.09
Mean	42.98	423.38	128.62	36.89	34.85	49.48	3.12	10.35
SD	1.07	9.89	3.65	3.39	3.21	10.38	3.27	1.71

FHD, femoral head diameter; L, femoral length; FNA, femoral neck angle; OAS, absolute offset; OSH, horizontal offset; OSV, vertical offset; GTH, greater trochanter height; ATA, anteversion angle.

The absolute relative differences (*δ*) between the resultant of the joint contact forces calculated by the musculoskeletal models and the resultant of the reaction forces produced by the finite element model were found to range between 2% and 10% among all models, as shown in Table 5. This shows that the boundary conditions of the finite element models were statically comparable to applying the appropriate hip and joint reaction forces.

**Table 5:** Data analysed for musculoskeletal and finite element models.

Case ID	JCF_hip (N)	JCF_knee (N)	JCF_net (N)	R_FEM (N)	%δ	e1	e3	%gait
1	2321	1880	747	779	4	0.32%	-0.35%	14
2	4323	2231	2225	2178	2	0.21%	-0.15%	47
3	4275	1831	2541	2659	5	0.37%	-0.67%	50
4	1689	1010	738	814	10	0.10%	-0.05%	19
5	3829	2584	1672	1585	5	0.21%	-0.20%	45
Mean	3287	1907	1585	1603	-	0.24%	-0.29%	-
SD	1207	586	829	829	-	0.11%	0.24%	-

Hip and knee joint contact forces (JCF) estimated by the musculoskeletal models, resultant of the reaction forces and the predicted first and third principal strains by the finite element models. % gait is the load step (out of 100 intervals) at which the peak principal strains were predicted.

JCF_hip, hip joint contact force calculated by the MSKM; JCF_knee, knee joint contact force calculated by the MSKM; JCF_net, resultant of the joint contact forces calculated by the MSKM; R_FEM, Resultant of the reaction forces in the FEM; %***δ*** is the absolute relative differences between the resultant of the joint contact forces calculated by the musculoskeletal models and the resultant of the reaction forces produced by the finite element model; e1, first principal strain; e3, third principal strain; %gait, is interval of the gait cycle at which the peak strains were predicted.

**Fig 4:**
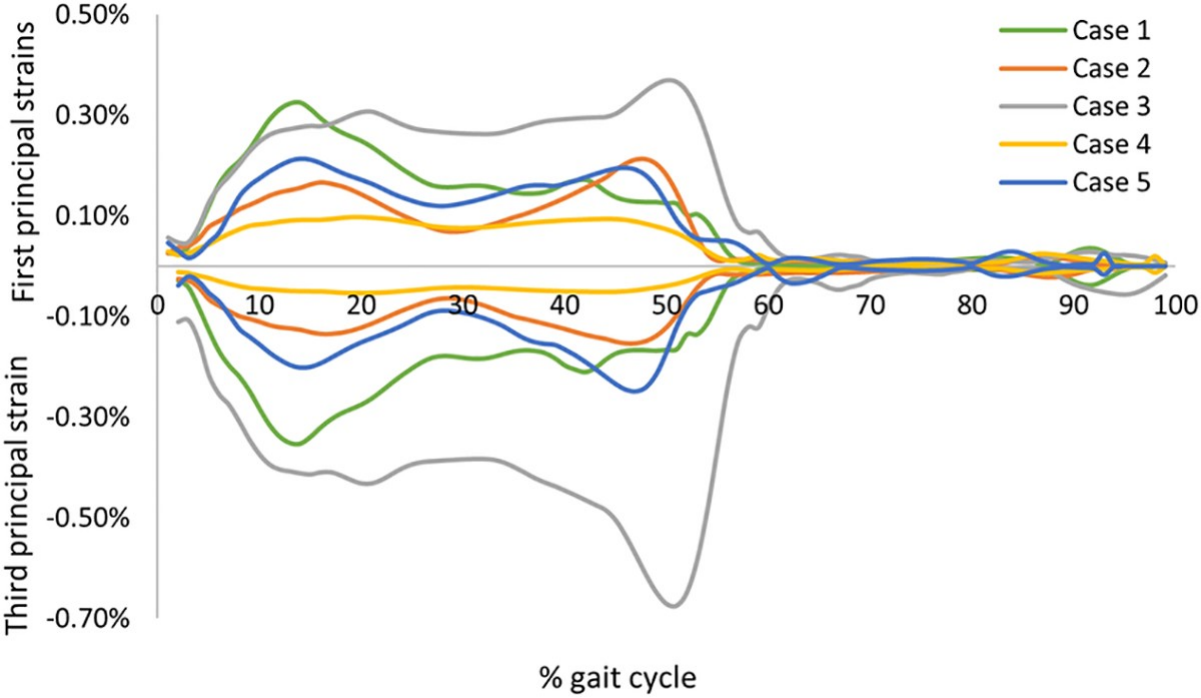
First and third principal strains predicted by the finite element models of the five cases. The peak strain values were predicted at each of the 100 intervals across one gait cycle.

The peak strains predicted at the femoral neck varied considerably among the five cases. The peak first and third principal strains ranged from 0.10% to 0.37% and -0.05% to -0.67%, respectively (Table 5). The predicted peak strains were lower than the fracture threshold of the human bone [44] for all cases, as expected. Case 3 had the highest peak predicted strains, while Case 4 had a noticeably lower peak strains in comparison to the other cases. The evolution of the maximum first and third principal strains predicted at the femoral neck along the full gait cycle for the five cases is shown in Fig 4. For four cases, two overall peaks of the strains were observed at around 15% and 50% of the gait cycle, while only one case (Case 1) had one peak at 15% of the gait.

**Fig 5:**
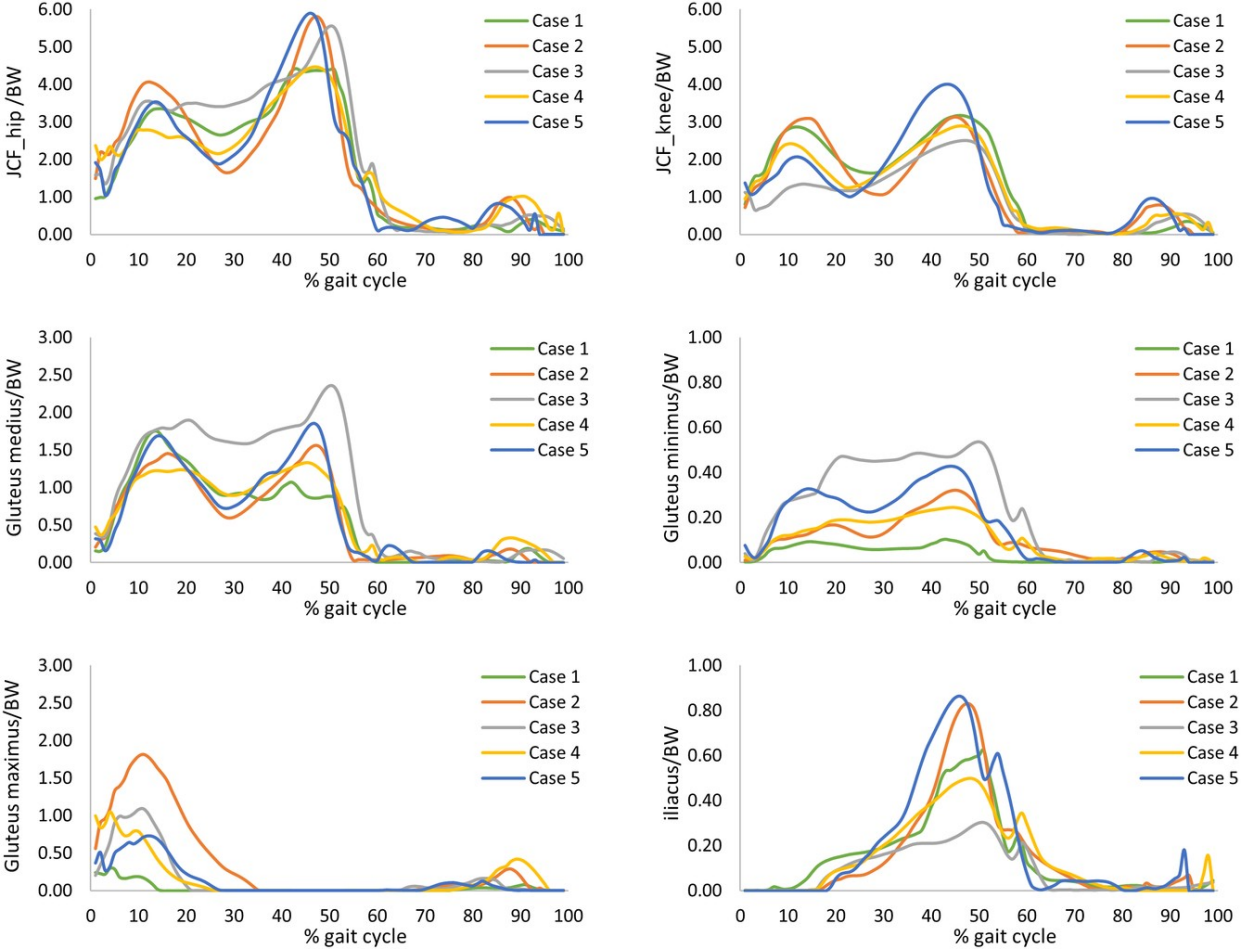
Hip and knee joint contact forces and muscles forces. A selection of the major muscles attached to the proximal femur as calculated by the musculoskeletal models along a full gait cycle for the five cases normalised by the body weight (BW).

**Fig 6:**
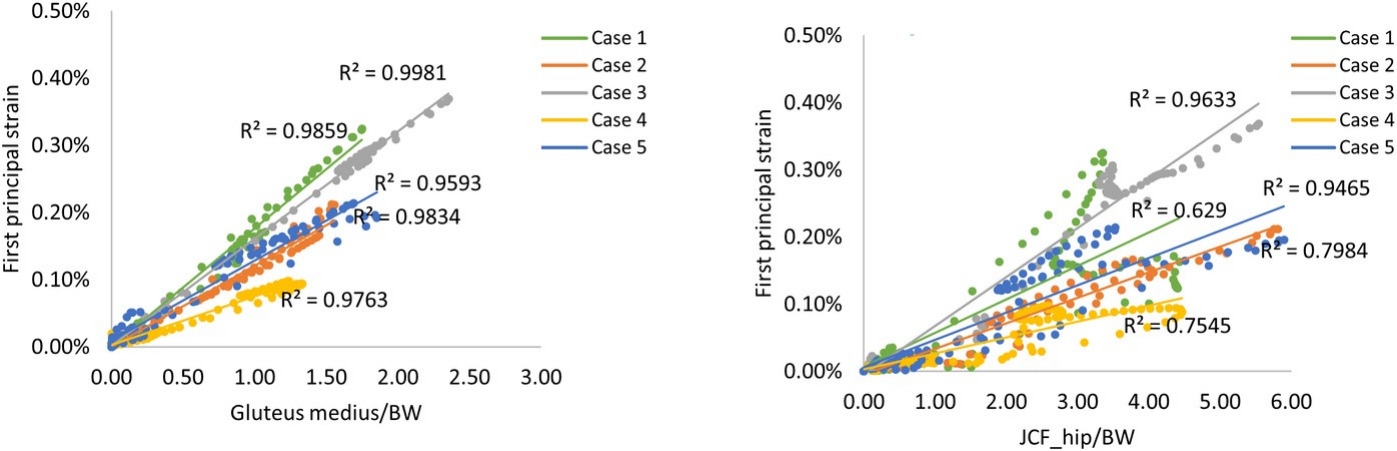
Correlation analysis. Correlation analyses performed for the peak first principal strain in the femoral neck and the gluteus medius muscle (left), and hip joint contact forces (right) acting on the femur during a full gait cycle normalised by the body weight (BW).

Fig 5 shows the joint contact forces at the hip and the knee, and the forces for the major muscles attached to the proximal femur along the gait cycle as predicted by the musculoskeletal models and normalised by the body weight. The gluteus medius muscle was found to have considerable effects on loading in the femoral neck during level walking in comparison to the other muscles investigated. A very strong correlation was found between gluteus medius muscle normalised by the body weight and the peak tensile strain in the femoral neck for the five cases with R^2^ value ranged between 0.96 and 0.99. While the correlation between the peak strains and hip joint contact forces normalised by the body weight varied from fair to good correlation among the cases with R^2^ ranged from 0.63 to 0.96 (Fig 6).

**Fig 7:**
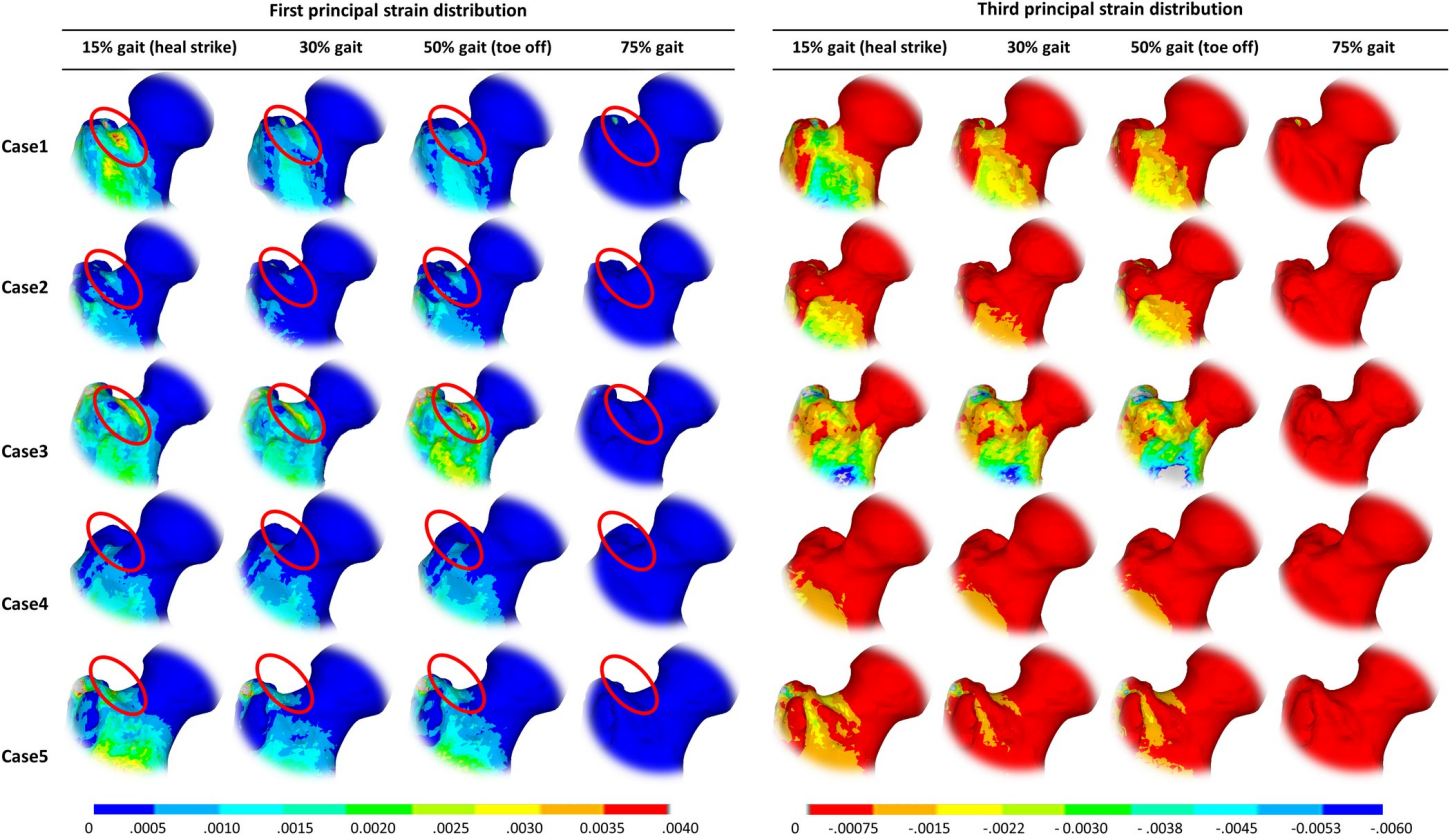
The overall First and third principal strain distribution within the femoral neck. Strains are shown at 15%, 30%, 50%, and 75% of the gait cycle for all the cases. The locations of the peak strains are indicated by the red circle.

**Fig 8:**
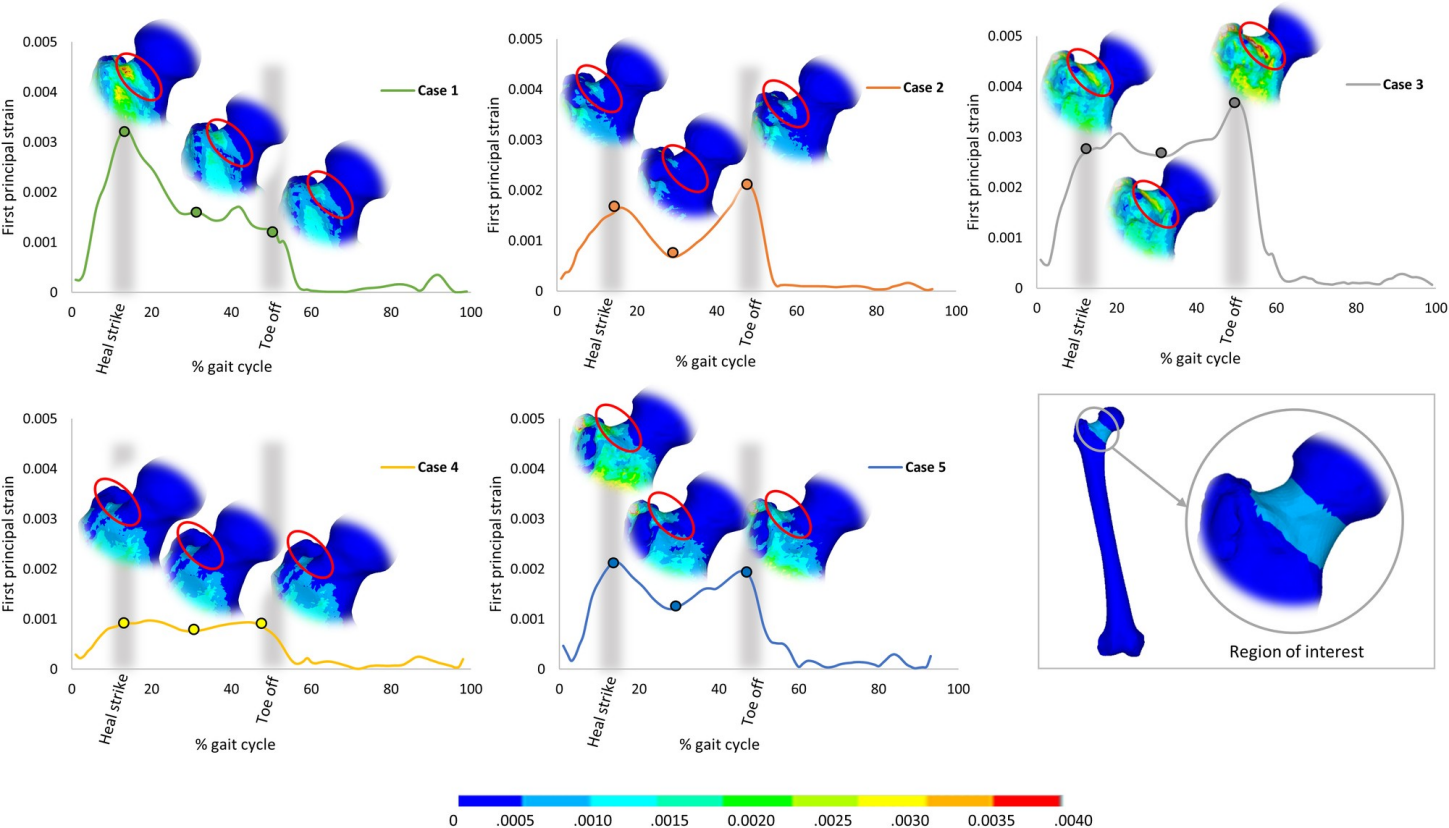
Peak first principal strain as predicted in the femoral neck at the 15%, 30%, and 50% of the gait cycle for all the cases. Heel strike and toe off, at which the two peaks of the principal strain are predicted, are indicated in grey shaded area. The location of the peak first principal strain region is indicated by the red circle. Region of interest at which the peak strains were estimated is indicated on the bottom right of the figure.

Failure was found to occur under tension for all the cases. Peak first principal strains were predicted at the superior neck region of the femur in the finite element model for all cases, as shown in Figs 7 and 8.

## 4 Discussion

The aim of the current study was to investigate the mechanical response of the femur during a normal gait cycle using a fully personalised coupled body-organ modelling approach. The muscle and joint contact forces [7–11] were calculated from multibody dynamics models, and the finite element method was used to predict the strains in the femoral neck [45, 46] during level walking. Five participants were included in this study in order to explore the variability of the predicted strain patterns among individuals.

Considerable variation was found in the predicted peak strain among individuals with a mean peak first principal strain of 0.24% ± 0.11% and a mean third principal strain of -0.29% ± 0.24%. Although the peak strain values were considerably different across the cases investigated in the current study, the predicted strain level was largely in agreement with previously reported findings in the literature [20, 22]. Martelli et al. [20] found that the peak tensile strain in the femoral neck during level walking is around 0.25% with one subject, while Kersh et al. [22] reported a peak tensile strain of about 0.16% as an average from twenty subjects.

The predicted peak strains for all cases were notably below the fracture threshold [44]. This finding supports the theory that, in the absence of trauma, bone fracture is only likely to occur when people with very weak bones undertake tasks that result in high loading conditions [21]. The highest peak strains were predicted for case 3, which could be an indication that this individual has slightly weaker bones than the other participants. However, case 3 has the shortest femur, the smallest femoral head, and the highest body weight among all cases with a T-score of -1.2. Future work should look into the bone mineral density distribution of this individual and the predicted strain under sideway fall loading condition in order to confirm this observation, and identify parameters that directly correlate to the weaker bone [47]. Such parameters could be measurements taken during level walking as well as geometrical features measurable around the proximal femur using imaging.

The range of measured morphological parameters of the femur for the five cases were found to be within the ranges reported in the literature [29, 48, 49]. The case that was predicted with the highest peak strain (Case 3) was found to have the smallest femoral neck angle (123.30^o^), while the case with the lowest predicted strains (Case 4) had a larger femoral neck angle (132.30^o^). These findings contradict what has been reported in the literature, where women with femoral neck fractures were reported to have wider femoral neck angles than women without femoral neck fractures [50]. Although it is generally accepted that femoral neck angle has a strong association with the fracture risk [50, 51], the authors’ opinions seemed to vary widely about using the femoral neck angle as a predictor for osteoporotic hip fracture [52]. Other studies have reported that bone geometry has a limited role in the load transmitted to the lower extremity compared to the soft tissue [53]. However, considering the small dataset of the current study, it was difficult to make a clear conclusion on the role of the femoral neck angle.

Our findings suggest that not only joint contact forces but also muscular forces substantially influence the loading at the femoral neck during a normal gait cycle. Four cases had two overall peaks of the maximum strains occurring when both feet were in contact with the floor (heel strike), i.e. when joint contact forces were at maximum, which agrees with what has been reported previously [22]. Nevertheless, one case (case 3) had only one peak maximum strains, at the toe off phase, despite having two distinct peaks in the hip joint reaction forces. Interestingly, the gluteus medius muscle of this particular case showed a similar trend to the predicted strain pattern with a single peak at the toe off phase. The marked contribution of the internal muscular loading on the femoral neck strain pattern is also confirmed by the excellent correlation found between the predicted peak strain and the gluteus medius muscle force. It has been reported that gluteus medius muscle induce high focal strains at the anterosuperior region of the femoral neck [22], which is in agreement with the predicted peak strain location for the five cases in the current study. This indicates that the activation of major muscles attached to the proximal part of the femur (in particular the greater trochanter) have a considerable contribution to the femoral neck loading during normal gait cycle.

With a standard (generic) approach, you would use experimental markers to scale a generic model to the subject anthropometry by measuring the ratio of the distance between couples of virtual markers (in the generic model) and experimental markers (on the subject). This scaling factor is applied to bones, including size and inertia properties, and to muscle geometry and properties. The benefits of using imaging information to personalise a MSK model were reported by many authors [54–56]. For instance, Bosmans et al. [54] showed how the difference between location of scaled and MRI-based muscle points can vary depending on subject’s characteristics and on the body segment. This difference can highly affect the accuracy of the MSK simulations by altering the resulting musculotendon parameters and the muscle moment arm. Correa et al. [57] and Scheys et al. [58, 59] observed differences over 30% of the moment arm between scaled generic and MRI-based models [57–59]. This can influence the calculation of muscle forces and joint contact forces, which are parameters used as inputs to the FE model. Although our modelling framework involves additional work in comparison to modelling based on a generic model, our proposed modelling framework can act as a benchmarks for future studies at which the generic model is only used. Future work can be focus on investigating the effect of using data based on generic model versus subject-specific MSK models on the femoral neck strain predicted by the FE model.

There are a few limitations to this study. First, although the number of subjects used reflects a substantial improvement from previous studies (mostly using a single subject), it is still insufficient to allow a full exploration of intra-personal variations. It has been stated that population-based studies are important to understand if individual anatomical parameters, bone quality, and motion patterns may lead to different strain levels and strain patterns [14]. The five subjects of the current study illustrated the necessity of considering a much wider cohort to capture the variability in the strain level and strain pattern of the femoral neck among individuals.

Second, the study focused on one gait cycle while the gait pattern of an individual might differ in two sequential gait cycles [60], producing different joint, muscle and ground reaction forces, and therefore inducing different strain levels and patterns in the femoral neck. However, a ten-meter-long walkway was considered during the gait data collection to ensure a natural cadence of the individual while walking, and hence minimising variations. Furthermore, the investigation of the gait variability was beyond the main aim of the current study.

A third important limitation is that the muscle activations were predicted assuming optimal neuromuscular control; as we age a combination of neurological, sensorial, and anatomo-functional changes tend to make this more unlikely. Previous studies from Martelli et al. [13] and Van Veen et al. [61] showed that a suboptimal control can increase considerably the loading on the skeletal joints.

This manuscript describes a fully personalised body-organ multiscale modelling approach, which could be later used to investigate other applications (e.g. loading behaviour at the distal femur close to the knee joint) if appropriate input data is available. Perhaps, the most vital limitation for this kind of work is validation. Previous studies focused on the validation of models at each scale independently. CT based finite element models have been very well validated and were reported to be able to predict bone strength and fracture onset with excellent accuracy compared with experiments [1, 5, 45, 62]. While musculoskeletal models have been validated with motion tracking data and the corresponding in vivo data of the hip joint [63–65]. However, quantification of the accuracy of coupled models is challenging. One possible solution is to leverage on the recent development of various verification, validation and uncertainty quantification (VVUQ) tools to aid the validation process.

In conclusion, the current study proposed a procedure for a fully personalised multiscale (body-organ level) model to investigate the femoral neck loading during a normal gait cycle. The model can be extended to be used for various applications (e.g. orthopaedics, where this modelling approach could help planning treatment for hip and knee replacement). The current findings also suggest that personal variations are substantial. Therefore, it is important to have subject-specific data and multiple subjects should be studied before deriving general conclusions for a target population.
